# Effects of physical exercise on muscle metabolism and meat quality characteristics of Mongolian sheep

**DOI:** 10.1002/fsn3.2768

**Published:** 2022-03-01

**Authors:** Min Zhang, Yueying Guo, Rina Su, Mirco Corazzin, Jiale Li, Huan Huang, Yue Zhang, Duo Yao, Lin Su, Lihua Zhao, Ye Jin

**Affiliations:** ^1^ College of Food Science and Engineering Inner Mongolia Agriculture University Hohhot China; ^2^ Inner Mongolia Vocational College of Chemical Engineering Hohhot China; ^3^ Dipartimento di Scienze Animali Università di Udine Italy

**Keywords:** meat quality, mongolian sheep, muscle metabolism, physical exercise

## Abstract

The objective of this study was to investigate the effects of exercise training on muscle metabolism, fatty acid composition, carcass traits, and meat quality characteristics of Mongolian sheep. Fourteen Mongolian sheep were randomly divided into two groups (7 sheep in each) and placed in two adjacent livestock pens. One group of sheep was kept in the pen (Control [C] group) and the other group of sheep (Training [T] group) were driven away in a field to walk twice a day. The results showed a reduction in pH measured 45 min post mortem, L*, a*, and b* value, intramuscular fat, and carcass length, and an increase in the ultimate pH value and shear force in the meat of T group in comparison with that of C group (*p *< .050). Also, exercise training moderately affected the fatty acid composition of LT muscle. Compared with C group, the concentrations of myristoleic acid (C14:1) and stearic acid (C18:0) were increased (*p *< .050), while the concentrations of C20:3 n‐6, neurolic acid (C24:1), and n‐3 polyunsaturated fatty acid (PUFA) were decreased in T group (*p *< .050). Transcriptome analysis highlighted 621 genes differentially expressed in two groups, including 385 were up‐regulated (e.g., *GLUT4* and *PGC‐1α*) and 236 were down‐regulated (e.g., *PLIN1* and *ACSL3*) in T with respect to C group. Besides, considering these genes, a number of enrichment pathways related to muscle metabolic processes, involving carbohydrate metabolism, lipid metabolism, oxidation reduction process, and muscle tissue development, were highlighted. In conclusion, these results contributed to a better understanding of the possible biological and molecular processes underlying the effects of exercise training on muscle metabolism and meat quality in Mongolian sheep, and provide useful information for contributing to understand the phenotypic and functional differences in meat quality of sheep.

## Graphical abstract

Three months of exercise training effectively changed the muscle metabolism process of Mongolian sheep, resulting in different degrees of influence on carcass characteristics, meat quality, and fatty acid composition. These findings provide insight into the mechanism by which exercise training affects the quality traits and fatty acid composition of Mongolian sheep, and contribute to a better understanding of muscle physiology and the biological and molecular processes of meat quality.Graphical Figure

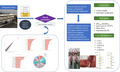

## 1 INTRODUCTION

Producing mutton with desirable eating quality is a key goal of the mutton industry. The quality and nutritional value of meat are important factors for consumer satisfaction and willingness to purchase (Jiang et al., 2015). With the increasing importance of grassland protection and the change in Chinese policies, sheep feeding has gradually evolved from natural grazing to centralized feed farms, which inadvertently limits the amount of physical activities that sheep can partake. The increased physical activity can lead to leaner meat with an increase in polyunsaturated fatty acids (PUFA); conversely, desirable sensory traits tend to increase with fat (Wood et al., 2008). Thus, the current production system is receiving increasing attention by consumers, and how animals are raised determines what they buy at the retail counter (Grunert et al., 2004).

Exercise activities, which consume a large amount of energy, require a metabolic recovery process to restore substrate stores, and a plethora of metabolic changes occur in order to regain substrate homeostasis, especially in previously active skeletal muscles (Lundsgaard et al., 2020). Skeletal muscle participates in the metabolism of fat and glucose at the same time (Yu et al., 2016), and these two metabolisms will affect each other under different exercise programs (Bonen et al., 2006; Coyle et al., 1997; Ranallo & Rhodes, 1998). Muscle storage of triglycerides is believed to be a major source of free fatty acids oxidized during physical exercise (Ranallo & Rhodes, 1998; Romijn et al., 1993). As the result of the long‐term physiological metabolism process of muscle, the determination of meat quality is related to the metabolic characteristics and development state of muscle during growth. Aalhus et al. (1991) found that the muscles of exercised sheep were significantly tender than muscles of their control counterparts due to a larger myofibrillar protein to total collagen ratio and a slightly higher pH at post mortem resulting from exercise. Gangnat et al. (2017) forced the calves to perform different physical activities, enough to cause muscle metabolic adaptation and make a difference in meat quality. Thus, the animals’ physical activity during the preslaughter phase, which is closely related to the growth state, has a key practical significance to muscle metabolism and meat quality.

The aim of this study was to assess the effect of exercise training on fatty acid composition, carcass performance, and meat quality of Mongolian sheep by considering RNA‐seq analysis to highlight key relevant biological networks and genes associated with muscle physiology. The hypothesis was that long‐term exercise training can induce adaptive physiological responses in sheep, improving carcass traits and meat quality. Elucidation of the underlying mechanisms could help us to seek novel approaches to optimize the way of feeding in the livestock industries.

## 2 MATERIALS AND METHODS

The experiments were conducted by the National Institute of Animal Health, China (GB 14,925–2001) and the Inner Mongolia Agriculture University Animal Care (Permit number: IMAU‐2017323) according to the guidelines.

### 2.1 Animals

A total of 14 Mongolian sheep from Inner Mongolia (China) were raised in the same environment with natural light and randomly assigned to two homogeneous experimental groups, control (C) and exercise training (T), according to body weight (21.19 ± 0.75 kg) and age (3 month). Each experimental group was composed of three rams and four ewes. Rams were castrated at birth. The trial lasted 3 months after an adaptation period of 1 week. During the adaptation and experimental period, C and T groups were kept in two adjacent pens of the same size (20 m^2^) in a feedlot, but during the experimental period, the animals belonging to T group were driven away in a field of 18.5 m × 10.5 m to walk twice a day (5 hr interval), 40 min each time. The distance and steps covered by each animal were monitored with a pedometer. During the experimental period, each animal in T group covered an average daily distance of 6000 ± 200 meters with 10,000 ± 500 steps. During the adaptation and experimental period, all the animals received the same diet which was based on concentrated feed and corn concentrate, and they had free access to water and hay. Animals were fed collectively within the exercise training group and the control group separately in two pens, and particular care was taken to ensure that all animals can have simultaneous access to feed.

At the end of the experimental period, all the sheep were slaughtered the same day. In particular, after an on‐farm fasting period of 24 hr, and the recording of the live weight for the calculation of the total body weight gain, the animals were transported to the abattoir (Bayannur Agriculture and Animal Husbandry, Inner Mongolia, China) and immediately slaughtered according to standard commercial “*halal*” procedures. The abattoir was 50 km away from the farm.

### 2.2 Carcass characteristics, samples collection, and meat quality evaluation

Determination of carcass characteristics referred to the method proposed by Valadez‐García et al. (2021). After slaughter, the carcass weight, carcass length, and thorax depth were measured. Within 30 min of exsanguination, the left side of the carcass was ribbed between the 12th and 13th rib to measure the *longissimus thoracis* (LT) m. area (dot square grid) and fat thickness (vernier caliper) and, from the three carcasses of randomly selected each experimental group, samples of approximately 5 g were collected, immediately snap frozen in liquid nitrogen, and stored at −80°C until total RNA extraction. The LT muscle between the 12th and 13th rib was also used to evaluate meat quality traits. Muscle pH was measured at 45 min and 24 hr post mortem using a PB‐10 portable pH meter. The meat color was measured within 30 min of bloodletting, and a chromatometer (Konica Minola, Japan) was directly located on the whole meat surface to measure lightness (L*), redness (a*), and yellowness (b*). The average of three measurements was recorded. Approximately 350 g of LT muscle was collected within 30 min of exsanguination and divided into three portions. The first portion, (20 g), was chopped in a commercial mixer blender, stirred well, and intramuscular fat (IMF) content was analyzed by Soxhlet (GB 5009.6–2016, AOAC, 2000). The second portion (5 g) was used for fatty acids analyses. The third portion (300 g) was chilled in a chiller at a mean temperature of 4 ℃ for 24 h for subsequent cooking loss determination and shear force measurement. The samples were weighed before (W1, g) and after (W2, g) 45 min of heating in a water bath until the central temperature reached 75°C in sealed bags, and cooking loss (%) was calculated as *(W1‐W2)/W1 × 100*. Then, the cooled sample from heating was measured as the shear force (kg/cm^2^) using a CL‐M muscle shear force measuring instrument (Jingke, China) for determination of the tenderness of the muscle. More than 10 rectangular cores (1 cm^2^) parallel to the longitudinal direction of muscle fibers were taken for analysis. The maximum and minimum values of each sample were removed, and the final shear force value was obtained by averaging the remaining values.

### 2.3 Fatty acid composition

The total lipids in the samples were extracted according to Folch et al. (1957). Five grams of minced LT muscle were put into extraction solvent (chloroform/methanol; 2:1, V/V), filtered, mixed with saline solution (20% sodium chloride), and put into separation funnel. After mixing and separation, the aqueous methanol portion was discarded, and the chloroform lipid portion was retained. The lipid extract obtained by additional filtration and rotary evaporation was transferred and methylated with hexane (1 ml) and 0.5 N methanolic KOH (3 ml) tubes for subsequent gas chromatographic analysis. Fatty acid methyl esters were injected (1 μl) into a TR‐FAME fused silica capillary column (thermo, 100 m × 0.25 mm i.d. × 0.25 μm film thickness) in the Thermo 1300 GC, and then detected by Thermo ISQ ion trap mass spectrometer (MS) operating in full‐scan mode. The specific operating conditions were as follows: the temperature program was initially set at 120℃ for 3 min; then, the temperature program was set at 195℃ for 2 min at a speed of 4℃ / min; and then, it was raised to 220℃ at a rate of 2℃ / min and maintained for 25 min. The injector temperature was maintained at 250℃, its volume was 1 μl; helium was used as carrier gas and the constant flow rate was 1 ml / min; the split ratio was 100:1, the ion source temperature was 250℃, and the scanning mass range of 40–450 m / z was obtained. According to the standard identification of Sigma Aldrich (Diesenhoff, Germany), the fatty acid composition is expressed as the percentage of total fatty acid methyl ester based on the similar peak retention times of standard.

### 2.4 Total RNA extraction, transcriptome, and qRT‐PCR analyses

Total RNA of the muscle samples was extracted using TRIzol^®^ Reagent according to the manufacturer's instructions (Invitrogen, California), and genomic DNA was removed using DNase I (TaKara, China). The integrity of RNA was verified by agarose gel electrophoresis and RNA quality was determined by 2100 Bioanalyser (Agilent, America) and quantified by the ND‐2000 (NanoDrop Technologies, America). Only high‐quality RNA sample meeting the conditions (OD 260/280 = 1.8–2.2, OD 260/230 ≥ 2.0, RIN ≥6.5, 28S:18S ≥ 1.0, >1 μg) could be used to construct sequencing library.

Transcriptome analysis was carried out by Majorbio (Shanghai Majorbio Bio‐pharm Technology, Ltd, China). One microgram total RNA was used to prepare the RNA‐seq transcriptome library using the TruSeq^TM^ RNA sample preparation kit from Illumina (America). The mRNA was first isolated by oligo (dT) beads polyA selection and then fragmented with fragmentation buffer. Secondly, double‐stranded cDNA was synthesized using SuperScript double‐stranded cDNA synthesis kit (Invitrogen, America) with random hexamer primers (Illumina, America) and according to Illumina's library construction scheme; the synthesized cDNA was subjected to end‐repair phosphorylation and “A” base addition. Libraries were size selected for cDNA target fragments of 300 bp on 2% low‐range ultra agarose followed by PCR amplified using Phusion DNA polymerase (NEB) for 15 PCR cycles. Thirdly, the Illumina HiSeq xten/NovaSeq 6000 sequencer (2 × 150bp read length) was used to sequence paired‐end RNA‐seq sequencing library after quantified by TBS380.

The raw paired‐end reads were trimmed and quality was controlled by SeqPrep 9 ) and Sickle (https://github.com/najoshi/sickle) with default parameters. Then, clean reads were separately aligned to reference genome using HISAT2 (http://ccb.jhu.edu/software/hisat2/index.shtml) software and the mapped reads of each sample were assembled by StringTie (https://ccb.jhu.edu/software/stringtie/index.shtml?t=example). The expression levels of the genes were quantified by the RSEM (http://deweylab.biostat.wisc.edu/rsem/) and, after obtaining the read counts of genes, DESeq2 was used to analyze the gene differential expression to identify the differentially expressed genes (DEGs; |log2FoldChange| ≥ 1; *p *< .050).

For RT‐PCR analyses, 2.0 μg RNA (500 ng μl^−1^) of each sample was used for cDNA synthesis using a PrimeScript^TM^ RT Reagent kit with gDNA Eraser (Perfect Real Time) (Takara, China) according to the manufacturer's instructions. For real‐time polymerase chain reaction (RT‐PCR), the reaction was performed on a Light Cycler 480 Ⅱ (Roche Diagnostics, Germany) in a 25 μl volume including 12.5 μl TB Green^®^ Premix Ex Taq^TM^II (Tli RNaseH Plus) (Takara, China), 8.5 μl of nuclease‐free water, 2.0 μl of forward and reverse primers (10 μM), and 2.0 μl of template cDNA. The RT PCR conditions were as follows: preincubation of 95°C for 30 s; 40 cycles of 95°C for 5 s, 57°C for 30 s, and 72°C for 30 s; melting of 72°C for 600 s, 65°C for 60 s, and 97°C for 1 s; and cooling of 37°C for 30 s. Melt curves were checked for excluding possible nonspecific amplification and primer–dimer formation.

The 18S gene was used as a stable reference house‐keeping gene in qRT‐PCR analyses. Moreover, statistical analysis of the threshold cycle values revealed that 18S gene was similarly expressed between experimental groups (*p *> .05). Each sample was analyzed in triplicate and the comparative 2^−ΔΔCt^ method was used for the relative gene expression (Livak & Schmittgen, 2001). In order to assess that primers efficiency was close to 100%, standard curves obtained by serial dilution of the pooled cDNA were considered (Pfaffl, 2001). All primers for qRT‐PCR are listed in Table S1

### 2.5 Statistical analysis

All data are expressed as the means ± *SEM*. Normality of data distribution was tested using the Shapiro–Wilk test. The differences between variables were analyzed by using a one‐way analysis of IBM SPSS Statistics 19.0 with the experimental group treated as fixed factor. Differences were identified as statistically significant at *p *< .050, while a *p *< .100 identified a trend toward statistical significance. The correlation between variables was assessed with Pearson's correlation test.

## 3 RESULTS

### 3.1 Animal performances, carcass traits, and meat quality

All sheep showed similar body weight at slaughter (*p *> .100). The carcass length in the exercise training group was significantly (*p *= .045) lower than in the control group and a trend for a lower (*p *= .090) total body weight gain was also observed in the T group, while no effects (*p *> .100) on any other carcass characteristics were found (i.e., final body weight, carcass weight, thorax Depth, fat thickness, and MLT area; Table 1). In comparison with the C group, the value of ultimate pH (*p *= .003) and shear force (*p *= .001) was increased, as well as the pH_45min_, the L* (*p *= .004), a* (*p *= .019), b* (*p *= .003) value, and intramuscular fat (IMF) (*p *= .001) were decreased in LT muscle of the T group (Table 2). There were no significant differences in cooking loss between the two groups (*p *> .100) (Table 2). The correlation results of various meat quality indicators are shown in Table 3. It is worth noting that the content of IMF had a significant negative correlation with shear force (r = −0.915), and a significant positive correlation with muscle color (r _L*_ = 0.948, r _a*_ = 0.891, r _b*_ = 0.853). pH_24h_ has a significant negative correlation with b* value (r = −0.736) and IMF (r = −0.782) and a significant positive correlation with shear force (r = 0.748).

**TABLE 1:** Effects of physical exercise training on carcass characteristics of Mongolian sheep

Items	Treatments		*SEM*	P‐values
	Control (C)	Training (T)^a^		
Final body weight (kg)	37.42	34.90	0.927	0.189
Total body weight gain (kg)	15.67	13.77	0.563	0.090
Carcass weight (kg)	17.76	17.60	0.451	0.871
Carcass length (cm)	74.75	72.75	0.526	0.045
Thorax Depth (cm)	18.71	19.00	0.206	0.510
Fat thickness (mm)	4.94	5.22	0.304	0.672
MLT area (cm^2^)^b^	13.00	13.24	0.514	0.832

Abbreviation: *SEM*, standard error of the mean.

^a^ Exercise twice a day, 40 min each time, 10,000 ± 500 steps and 6000 ± 200 meters of distance were required each day.

^b^ MLT area = longissimus *thoracis* m. area.

**TABLE 2:** Effects of physical exercise training on meat quality of the *longissimus thoracis* (LT) m. of Mongolian sheep

Items	Treatments		*SEM*	P‐values
	Control (C)	Training (T)^a^		
pH_45min_	6.30	6.14	0.041	0.046
pH_24h_	5.31	5.56	0.047	0.003
L^*^	35.73	31.73	0.816	0.004
a^*^	19.82	18.15	0.385	0.019
b^*^	3.91	2.93	0.196	0.003
Cooking loss (%)	33.72	35.19	0.545	0.193
Shear force (*N*)	43.35	81.66	6.808	0.001
Intramuscular fat (%)	3.41	2.98	0.089	0.001

Abbreviation: *SEM*, standard error of the mean.

^a^ Exercise twice a day, 40 min each time, 10,000 ± 500 steps and 6000 ± 200 meters of distance were required each day.

**TABLE 3:** Correlation among meat quality of the *longissimus thoracis* (LT) m. of Mongolian sheep

	pH_45min_	pH_24h_	L*	a*	b*	CL^a^	SF^b^	IMF ^c^	MLT area^d^
pH_45min_	1								
pH_24h_	−0.515	1							
L*	0.037	−0.395	1						
a*	0.219	−0.302	0.875**	1					
b*	0.310	−0.736*	0.916*	0.943**	1				
CL^a^	0.486	−0.099	0.008	0.232	0.117	1			
SF^b^	−0.663	0.748*	−0.765*	−0.580	−0.796*	−0.412	1		
IMF^c^	0.501	−0.782*	0.948*	0.891*	0.853*	0.742	−0.915*	1	
MLT area^d^	0.395	0.202	−0.497	−0.093	−0.055	−0.275	0.229	−0.032	1

^a^ CL = cooking loss (%).

^b^ SF: shear force (kg).

^c^ IMF: intramuscular fat (%).

^d^ MLT area = longissimus *thoracis* m. area (cm^2^).

* *p* < .05

** *p* < .01.

### 3.2 Fatty acid composition

The fatty acid composition in the LT muscle of Mongolian sheep is shown in Table 4. The proportions of myristoleic acid (C14:1) (*p *= .035) and stearic (C18:0) (*p *= .049) in the T group increased significantly, and dihomo‐gamma‐linolenic acid (C20:3n‐6; *p *< .001) and nervonic acid (C24:1) (*p *= .018) decreased significantly in comparison with the proportions in the LT muscle of C group. A trend for a higher myristic acid (C14:0; *p *= .058) percentage and a lower arachidonic acid (C20:4n‐6) percentage was also observed in the sheep after physical exercise training. Except that, there were no differences (*p *> .100) in other fatty acids between the two groups. In addition, the sum of n‐3 polyunsaturated fatty acids (PUFA; *p *< .001) in the T group was significantly lower than that in the C group, and a trend for higher (*p *= .095) n‐6/n‐3 ratio in the T group was found.

**TABLE 4:** Effects of physical exercise training on fatty acid composition (g/100 g FAME) in the *longissimus thoracis* m. of Mongolian sheep

Items	Treatments		*SEM*	P‐values
	Control (C)	Training (T) ^a^		
C10:0	0.14	0.12	0.009	0.284
C12:0	0.13	0.12	0.007	0.502
C14:0	1.91	2.27	0.096	0.058
C14:1	0.21	0.39	0.043	0.035
C16:0	22.84	22.20	0.399	0.449
C16:1	2.54	2.41	0.048	0.201
C18:0	12.40	13.58	0.309	0.049
C18:1n−9 t	1.4	2.16	0.250	0.156
C18:1n−9*c*	41.99	42.52	0.489	0.618
C18:2n−6 t	0.26	0.23	0.038	0.708
C18:2n−6*c*	8.87	9.17	0.717	0.843
C20:0	0.14	0.12	0.012	0.491
C18:3n−6	0.34	0.24	0.046	0.305
C18:3n−3	0.61	0.46	0.045	0.106
C20:3n−6	0.44	0.14	0.051	0.000
C20:4n−6	4.08	3.23	0.228	0.055
C20:5n−3	0.34	0.27	0.024	0.140
C24:1	0.60	0.40	0.046	0.018
SFA	38.01	38.81	0.693	0.589
MUFA	46.83	46.75	0.674	0.960
PUFA	15.16	12.64	0.996	0.226
*n*−3^b^	0.98	0.69	0.055	<0.001
*n*−6^c^	14.18	11.95	0.962	0.272
SFA/UFA	0.62	0.64	0.018	0.574
PUFA/SFA	0.37	0.32	0.028	0.385
*n*−6/*n*−3	14.43	16.96	0.755	0.095

^a^Exercise twice a day, 40 min each time, 10,000 ± 500 steps and 6000 ± 200 meters of distance were required each day.^b^C18:3n‐3 + C20:5. ^c^C18:2n6 t + C18:2n6c + C18:3n‐6 + C20:3 + C20:4.

Abbreviations: MUFA, monounsaturated fatty acids; PUFA, polyunsaturated fatty acids; *SEM*, standard error of the mean; SFA, saturated fatty acids.

### 3.3 Summary of RNA‐seq data

The filtered reads were mapped to the sheep reference genome. In this study, we obtained 62,763,294, 61,950,202, 56,311,578, 5,933,396, 65,762,900, and 600,549,556 raw reads for C group (C1, C2, and C3) and T group (T1, T2, and T3), respectively (Table 5). A total of 54.23 GB clean data were obtained. The clean data of each sample were above 8.34 GB, and the proportion of Q30 base was more than 96.1%.

**TABLE 5:** Reads mapping summary

Sample	Raw reads	Clean reads	Total mapped	Multiple mapped	Uniquely mapped
C1	62,763,294	62,182,774	59,995,413(96.48%)	9,325,084(15.0%)	50,670,329(81.49%)
C2	61,950,202	61,468,790	59,173,876(96.27%)	7,178,922(11.68%)	51,994,954(84.59%)
C3	56,311,578	55,882,420	53,924,385(96.5%)	8,296,167(14.85%)	45,628,218(81.65%)
T1	59,233,396	58,785,936	56,810,365(96.64%)	7,624,231(12.97%)	49,186,134(83.67%)
T2	65,762,900	65,288,632	62,974,091(96.45%)	9,296,285(14.24%)	53,677,806(82.22%)
T3	60,054,956	59,642,598	57,451,192(96.33%)	8,151,912(13.67%)	49,299,280(82.66%)

Note

Three replicates of control group (C1, 2, and 3) and training group (T1, 2, and 3) were carried out in RNA‐seq analysis.

### 3.4 Transcriptome analysis and identification of DEGs

**FIGURE 1:**
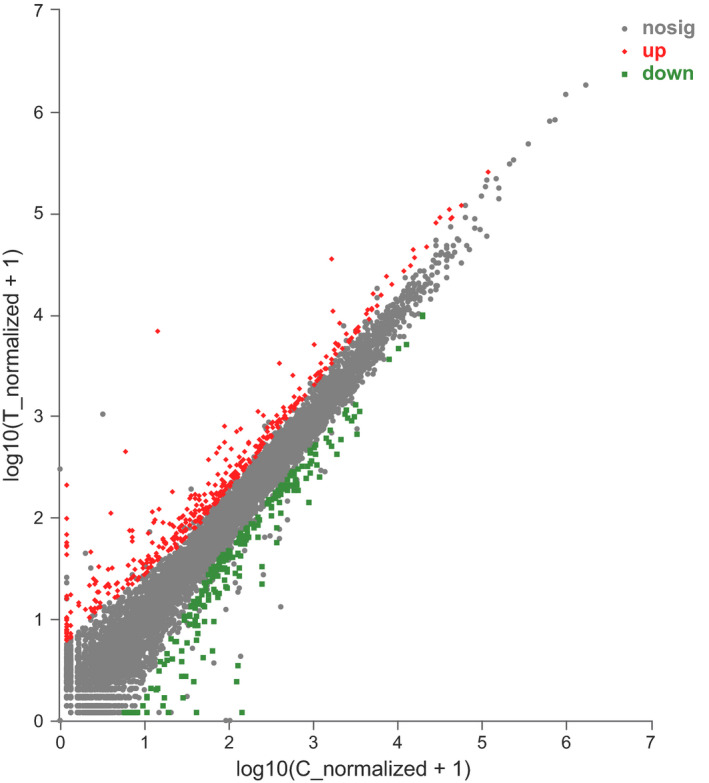
The differentially expressed genes (DEGs) in *longissimus thoracis* (LT) m. between the control group and the training group. Red dots (up) represent the 385 significantly up‐regulated genes (|log2FoldChange| ≥ 1; *p *< .050), and green dots (down) represent the 286 significantly down‐regulated genes (|log2FoldChange| ≥ 1; *p *< .050) in training group. Black dots (nosig) represent insignificantly differential expressed genes

Compared with the C group, there were 621 DEGs in the LT muscle of T group (Figure 1, Table S2), including 385 up‐regulated genes, such as *OLR1*, *PPARGC1A (PGC‐1α),* and *SLC2A4 (GLUT4)*, and 236 down‐regulated genes, such as *PIK3CD*, *PLIN1,* and *ACSL3*, in the LT muscle.

### 3.5 Gene Ontology (GO) and Kyoto Encyclopedia of Genes and Genomes (KEGG) pathway analyses

In total, 621 DEGs were identified in both groups according to three GO categories: biological process, cellular component, and molecular function. A total of 841 GO terms related to xenobiotic metabolic process, developmental process, and lipid metabolic process were revealed, 170 GO terms of chemokine activity, signaling receptor binding, and catalytic activity were found by significant analysis, and 48 GO terms of extracellular region part, myofibril, and plasma lipoprotein particle were found as well (Table S3). The pathway annotation of DEGs was performed by the Kyoto Encyclopedia of Genes and Genomes (KEGG) pathway and most KEGG pathways are involved in metabolism. In addition, the results showed that 39 pathways were significantly (*p *< .050) enriched in T group among all DEGs (Table S4).

**FIGURE 2:**
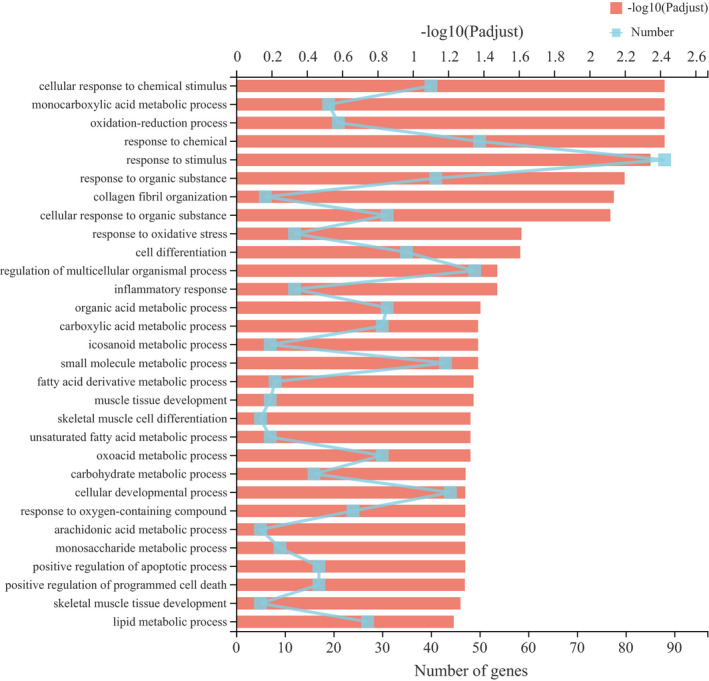
The top enriched (Adj *p *≤ .060) biological process terms in Gene ontology (GO) analysis of DEGs (up‐regulated) between the control and training groups

Significantly up‐ and down‐regulated blocks of functionally related genes were identified to explore the influence mechanism of exercise on muscle metabolism of Mongolian sheep. For this purpose, we selected the significant enrichment (adj *p* ≤ .060) GO terms satisfying GO ≥level 5 and for which at least five genes were identified for analysis (Table S5). Interestingly, none of the down‐regulated genes satisfy the GO terms condition filtered by the above condition. Among the up‐regulated genes, the variety of cellular component terms, including collagen‐containing extracellular matrix, extracellular matrix, extracellular space, and extracellular region part was abundant (Adj *p* ≤ .050) categories and for molecular function, aromatase activity, chemokine activity, and binding‐related terms were significantly (Adj *p* ≤ .050) enriched in all the three comparisons. The top enriched (Adj *p* ≤ .050) biological process terms are presented in Figure 2. The figure indicates that the up‐regulated DEGs mainly influenced biological process such as muscle tissue development, unsaturated fatty acid metabolic process, and carbohydrate metabolic process. These processes are involved in muscle metabolism, which is affected by exercise. Moreover, we selected 10 GO terms (carbohydrate metabolic process, lipid metabolic process, oxidation reduction process, monocarboxylic acid metabolic process, organic acid metabolic process, developmental process, cell differentiation, regulation of cell death, fatty acid derivative metabolic process, and regulation of phosphate metabolic process) that are directly related to muscle metabolism and development in biological process, and 30 DEGs associated with these GO terms were obtained (Table 6).

**TABLE 6:** DEGs associated with metabolism and development in biological process by GO analysis

Gene symbol	Description	Log2FC	P‐values	Regular (C vs. T)	Enriched biological process
PGK1	Phosphoglycerate kinase 1	1.2	1.70E−03	up	1,3,4,5,6,7,10
TPI1	Triosephosphate isomerase 1	1.1	2.09E−03	up	1,4,5
GK	Glycerol kinase, transcript variant X1	1.2	7.41E−03	up	1
PLIN1	Perilipin 1	−1.1	1.15E−02	down	2
DGAT2	Diacylglycerol O‐acyltransferase 2	1.3	2.69E−02	up	1,2,6,9
ACSL3	Acyl‐CoA synthetase long chain family member 3	−1.0	2.98E−03	down	10
ACSF3	Acyl‐CoA synthetase family member 3, transcript variant X1	1.0	4.87E−03	up	2,4,5
PGC−1α	PPARG coactivator 1 alpha, transcript variant X1	1.3	8.98E−03	up	3,6,7,8,10
GLUT4	Solute carrier family 2 member 4	1.2	1.74E−03	up	6,7
SOCS3	Suppressor of cytokine signaling 3	2.3	4.77E−02	up	2,6,7,8,10
GOT1	Glutamic‐oxaloacetic transaminase 1	1.3	1.69E−06	up	1,5
GSTA1	Glutathione S‐transferase alpha 1	−1.7	1.00E−07	down	2,4,5,10
GADL1	Glutamate decarboxylase like 1	2.2	4.70E−06	up	5
CYP1A1	Cytochrome P4501A1, transcript variant X1	1.9	2.49E−04	up	2,4,5
SDS	Serine dehydratase	1.4	1.31E−02	up	1,4,5
SYNJ2	Synaptojanin 2	2.2	8.84E−07	up	2
AKR1B1	Aldo‐keto reductase family 1 member B	1.2	1.69E−03	up	2
HK2	Hexokinase 2	1.5	4.14E−05	up	1,4,5
DHDH	Dihydrodiol dehydrogenase	1.1	2.13E−04	up	1
PKM	Pyruvate kinase M1/2, transcript variant X2	1.4	3.78E−04	up	1,4,5
AFG1L	AFG1 like ATPase	1.1	2.89E−02	up	3
MRPS36	Mitochondrial ribosomal protein S36	1.1	5.53E−04	up	3,5
CYP2D6	Cytochrome P450 2D6	1.7	9.71E−05	up	2,3,4,5,10
LDLR	Low‐density lipoprotein receptor	1.0	1.26E−02	up	2,6
GPCPD1	Glycerophosphocholine phosphodiesterase 1, transcript variant X1	1.0	2.15E−04	up	2,6
FGFR3	Fibroblast growth factor receptor 3, transcript variant X2	−2.1	1.18E−04	down	6,7,8,10
GDF11	Growth differentiation factor 11	1.1	4.99E−02	up	6,8,10
ND2	NADH dehydrogenase subunit 2	−1.0	1.40E−02	down	3
Wnt11	Wnt family member 11	1.3	2.06E−03	up	6,7
CAMK2A	Calcium/calmodulin‐dependent protein kinase II alpha, transcript variant X1	1.1	1.54E−02	up	6,8

Note

Enriched biological process: 1. carbohydrate metabolic process; 2. lipid metabolic process; 3. Oxidation reduction process; 4. monocarboxylic acid metabolic process; 5. organic acid metabolic process; 6. developmental process; 7. cell differentiation; 8. regulation of cell death; 9. fatty acid derivative metabolic process; and 10. regulation of phosphate metabolic process.

**FIGURE 3:**
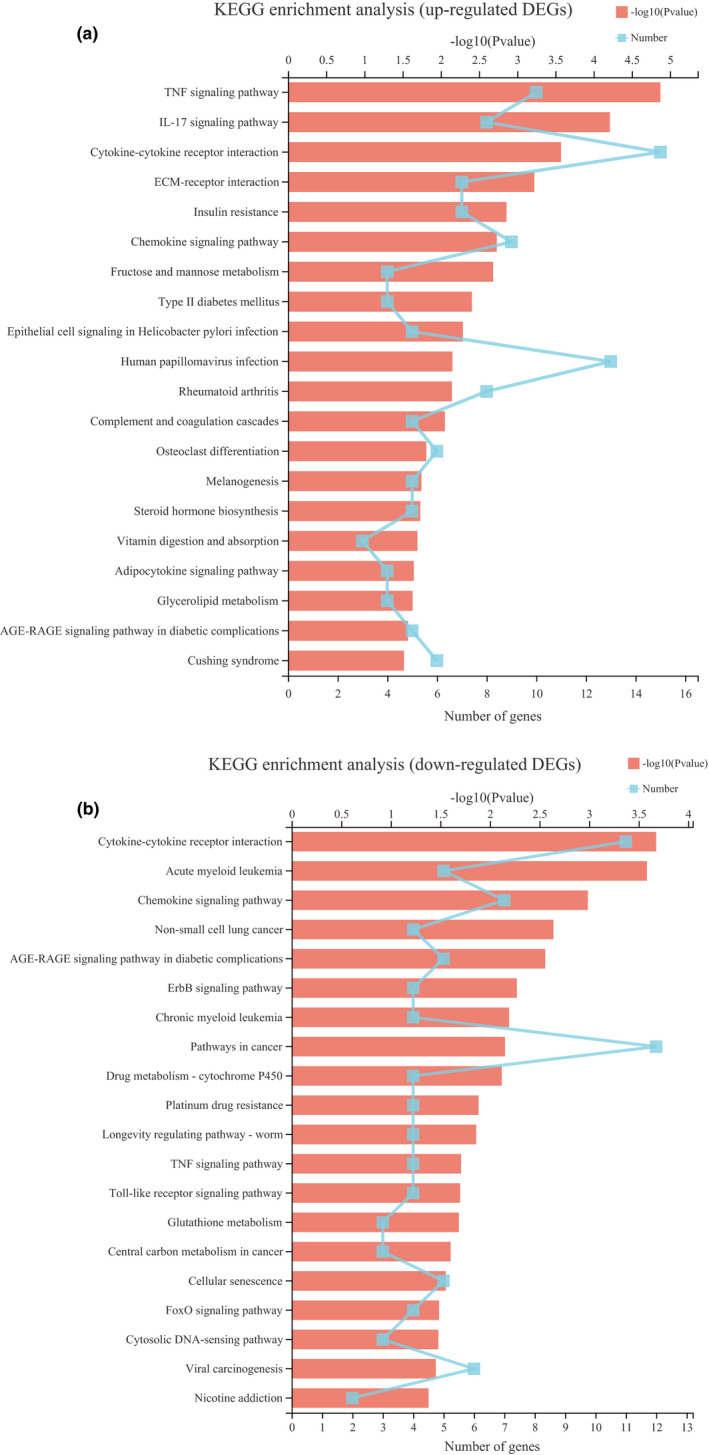
KEGG Pathway analysis of DEGs between the control group and the training group. (a)The significant enriched (*p* ___ 0.050) KEGG pathways for up‐regulated DEGs. (b)The significant enriched (*p*___ 0.050) KEGG pathways for down‐regulated DEGs

**FIGURE 4:**
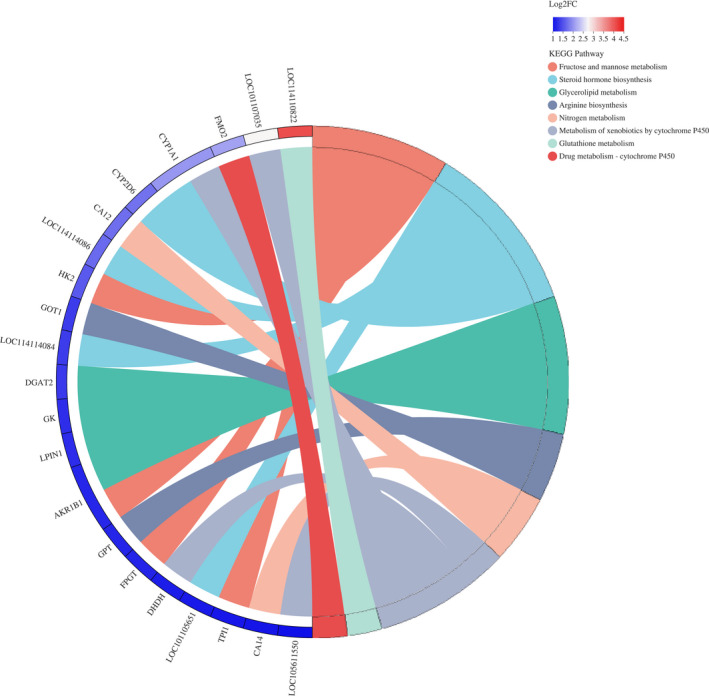
DEGs enriched in KEGG pathways related to the metabolic process. The genes are linked to their assigned terms via colored ribbons. Chords represent a detailed relationship between the expression levels of up‐regulated DEGs (left semicircle perimeter) and their enriched KEGG pathways (right semicircle perimeter)

The KEGG pathways significantly (*p *< .050) enriched for up‐ and down‐regulate are shown in Table S6. The top 20 significant pathways are shown in Figure 3a‐b. Pathway analysis showed that the up‐regulated DEGs are involved in carbohydrate metabolism, lipid metabolism, and amino acid metabolism, such as fructose and mannose metabolism, steroid hormone biosynthesis, glycerolipid metabolism, and arginine biosynthesis. The down‐regulated DEGs are mainly involved in human diseases and organismal systems. In further identification of the regulatory genes related to exercise affecting muscle metabolism, metabolic pathways were selected to analyze the enrichment of DEGs as shown in Figure 4. This indicated that these DEGs are involved in the mechanism by which exercise affects muscle metabolism.

### 3.6 Validation of RNA‐Seq data

**FIGURE 5:**
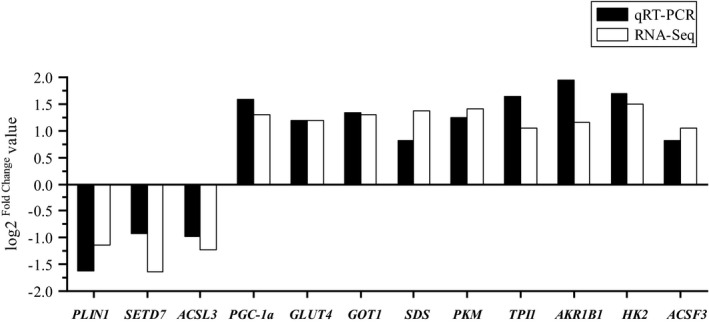
Validation of twelve differentially expressed genes by quantitative reverse transcription PCR (qRT‐PCR). 18S was used as an internal control, and data are presented as log2 Fold Change (*n* = 3 per group)

Twelve DEGs were selected randomly to validate the RNA‐Seq data by qRT‐PCR. As well as in RNA‐Seq analyses, *GLUT4*, *PGC‐1a*, *GOT1*, *SDS*, *PKM*, *TPI1*, *AKR1B1*, *HK2,* and *ACSF3* were significantly up‐regulated, while *PLIN1*, *SETD7,* and *ACSL3* were significantly down‐regulated (*p* < .050) in T in comparison to C group (Table S2). The expression levels of these genes determined by qRT‐PCR were consistent with the RNA‐Seq data, which validated the accuracy of the RNA‐Seq data (Figure 5).

## 4 DISCUSSION

### 4.1 Effects of exercise training on performances, carcass characteristics, and meat quality

Exercise is a common way to lose weight in humans, but the regulation of weight is complex because it is influenced simultaneously by genetic structure, the environment, and their interactions (S. A. Kelly et al., 2011). Our results showed that the exercise group tended to decrease overall weight gain (Table 1). The observed tendency to decrease the overall weight gain was probably due to increased physical activity in the T group that was not accompanied by increased energy supplementation in diet.

The average ultimate pH found in the present study was lower than 5.7 and, therefore, can be considered sufficient to maintain an appropriate shelf life of meat (Mach et al., 2008). The glycogen level in muscle is inversely related to the ultimate pH value at slaughter (Pighin et al., 2014). In the T group, the ultimate pH was significantly higher than that of the C group, which was probably related to the greater consumption of glycogen produced during exercise. Vestergaard et al. (2000) also confirmed that among bulls in extensive production systems, there was a tendency that the animals with the lowest glycogen level also had the highest pH value of meat. The glucose metabolic process (BP GO:0,006,006) and regulation of glycogen catabolic process (BP GO:0,005,981) gene ontologies were overrepresented among genes that increased expression following training (Table S3). Therefore, to ensure meat with appropriate ultimate pH, it is necessary to pay particular attention to the energy level in the diet of sheep that exercise frequently, especially marketable animals that exercise frequently should not experience a period of malnutrition in the weeks before slaughter.

In our study, b* values were negatively correlated with pH_24h_, consistent with Su et al. (2019) and Page et al. (2001), who confirmed that the higher pH value caused by physical exercise could have led to darker meat. Livestock subjected to extensive and frequent physical activity, like grazing, could produce meat that is darker than that produced from indoor housed animals (Muir et al., 1998; Wang et al., 2018). Combined with these results, we confirmed that the higher pH value caused by physical exercise could have led to darker meat. Animals with increased activity have greater amounts of myoglobin than their sedentary or inactive counterparts (Lawrie, 1953) and pigmentation is positively correlated with the redness (a*) value. Surprisingly, in this trial, physical exercise reduced the a* value. Similar results were found by Vestergaard et al. (2000), who observed a higher pigmentation level, but a lower a* value in *longissimus dorsi* m. of bulls extensively reared compared to that of bulls intensively reared.

Tenderness is affected by multiple factors including proteolysis, connective tissue, muscle contractile state (Belew et al., 2003), and intramuscular fat content (Starkey et al., 2016). In comparison with the nonexercised pigs, the exercised pigs had a lower proportion of intramuscular fat (Enfalt et al., 2010). This result is consistent with our findings that exercise reduced fat content in muscle and increased shear force. The transcriptional data that glycerolipid metabolism (map00561; Figure 3) was significantly enriched and the lower intramuscular fat in T group proves that exercise depletes intramuscular fat by enhancing triglyceride metabolism. Moreover, collagen in muscle is highly correlated with meat tenderness (Dransfield et al., 2003). Aalhus et al. (1991) observed a significant reduction in shear forces in *semimembranes* and *vastus lateralis* m. in lambs exercised on a treadmill 5 days a week throughout the growing period, with an increase in myofibrillary protein per gram of muscle and a decrease in collagen. The LT muscle is not directly involved in exercise because it is a supporting muscle. Intramuscular connective tissue has a significant effect on the meat tenderness through the characteristic changes in the collagen in the endomysia and perimysia. The results of our transcriptome study showed the collagen‐containing extracellular matrix (CC GO:0,062,023; Table S5) and collagen fibril organization (BP GO:0,030,199; Table S5, Figure 2). GO ontologies were also overrepresented among genes that increased expression following training. Although collagen content and solubility were not assessed in this trial, the results suggest that exercise increased the metabolism of collagen and promoted the development of connective tissue, resulting in greater shear force.

### 4.2 Effects of exercise training on fatty acid composition

The fatty acids profile found in the skeletal muscle of the carcass may be altered when an individual's physical exercise changes for extended periods of time. Szabo et al. (2002) evaluated the use of fatty acids as substrates during physical activity in rabbits, and exercise treatment significantly increased the oleic acid ratio (C18:1n‐9) in both *longissimus dorsi* m. and *vastus lateralis* m., while it reduced C18:0 and C20:4n‐6 in *vastus lateralis* m. Gerlach (2014) demonstrated that exercise treatment reduced C24:1 in the *longissimus lumborum* m. of beef cattle. In addition, Quiles et al. (1999) observed that exercise also yielded a lower percentage of C20:4n‐6 and n‐3 PUFA in the *vastus lateralis* m., and this is consistent with our findings. The tendential higher C20:4n‐6 in C than in T group was in agreement with transcriptome data, where the arachidonic acid metabolic process (GO:0,019,369; Figure 2) was significantly up‐regulated in the T group. Thus, 3 months of exercise training alters fatty acid composition by mobilizing fatty acids and, in particular, reduces some PUFA levels. On the basis of understanding the changes in fatty acid composition caused by exercise, we should add substances with targeted regulation of fatty acids to the diet to improve the fatty acid composition of Mongolian sheep in a direction that is more beneficial for human consumption.

### 4.3 Effects of exercise training on muscle metabolism process

The metabolic mechanism in muscle is complex, and manipulation of muscle development, intramuscular fat deposition, and fatty acid composition for meat production is very important in sheep breeding. RNA‐seq is a technique developed to determine the expression level of the whole genome, allowing rapid and comprehensive determination of almost all transcripts of a species, thus extending the frontiers of animal genetics (Wickramasinghe et al., 2014). To investigate the potential mechanisms by which exercise affects muscle metabolism and meat quality, a transcriptome analysis of the mRNA expressed in the muscle tissues of two groups of Mongolian sheep was performed. After transcriptome analysis, 12 differentially expressed genes between the exercise and control group have been properly verified by quantitative polymerase chain reaction analysis, which proves that RNA‐seq is a powerful tool for studying gene expression of Mongolian sheep.

In GO terms, many overexpressed up‐regulated genes were significantly enriched in the metabolic pathway, suggesting that exercise training promoted the muscle metabolic process of Mongolian sheep. McGivney et al. (2010) also demonstrated that exercise training can effectively improve the underlying gene expression patterns of a wide range of genes involved in energy production and metabolism. Among the significantly differentially expressed genes, nine mitochondria genes were up‐regulated in the T group: *IMMT*, *MRPL36*, *MRPL47*, *MRPS36*, *MTFP1*, *LOC101119765*, *LOC114111248*, *LOC114112897,* and *LOC114113605* (Table S2). They are nucleo‐encoding genes involved in ribosomal protein and mitochondrial division, and in mitochondrial protein synthesis. Therefore, an increase in ribosomal protein and mitochondrial division would promote an increase in mitochondrial abundance. *IMMT* (inner membrane mitochondrial protein, transcript variant X1) has been reported to be a mitochondrial protein that affects the morphological structure and function of mitochondria (John et al., 2005). *MRPL36* (mitochondrial ribosomal protein L36), *MRPL47* (mitochondrial ribosomal protein L47, transcript variant X1), and *MRPS36* (mitochondrial ribosomal protein S36) belong to the *MRP* family of genes, which encode mitochondrial ribosomal proteins essential for mitochondrial protein synthesis. They play an important role in the oxidative phosphorylation system, and mutations in these genes cause reduced ATP production, leading to neuropathy, myopathy, and developmental disorders (O'Brien, 2002). *MRPL36* plays a crucial role in mitochondrial translation, which determines the speed of assembly of the respiratory chain (Prestele et al., 2009). The oxidation‐reduction process (BP GO:0,055,114), unsaturated fatty acid metabolic process (BP GO:0,033,559), carbohydrate metabolic process (BP GO:0,005,975), and lipid metabolic process (BP GO:0,006,629) GO ontologies (Figure 2) were also overrepresented among up‐regulated expression genes following training. This is in agreement with previously observed conclusion in fatty acid metabolism and fatty acid oxidation after exercise training (Holloszy et al., 1977; McGivney et al., 2010). Furthermore, *PGC‐1α*, *SLC2A4,* and *LDLR* were up‐regulated in DEGs (Table S2), *PGC‐1α* (PPARG coactivator 1 alpha, transcript variant X1, *PPARGC1A*) has emerged as a major regulatory factor of mitochondrial biogenesis and function, and thus becoming an important metabolic node (Fernandez‐Marcos & Auwerx, 2011) and *LDLR* (low‐density lipoprotein receptor) is a key receptor for maintaining cholesterol homeostasis in mammals (Go & Mani, 2012). *SLC2A4* (solute carrier family 2 member 4, also known as *GLUT4*) has a crucial role in whole‐body glucose homeostasis and the exercise of muscle cells, or the addition of insulin, causes *GLUT4* to move from intracellular location to the plasma membrane (Bryant et al., 2002). However, in the absence of muscle contraction and increased muscle glucose metabolism, increasing glucose delivery alone is insufficient to increase muscle glucose uptake. Moreover, when glucose transporter isoforms are selectively absent from skeletal muscle, muscle glucose uptake is almost completely weakened during contraction/exercise (Cushman & Wardzala, 1980; James et al., 1988; Wardzala et al., 1978). Exercise‐induced *GLUT4* translocation has also been observed in many human skeletal muscle studies (Marette et al., 1992; Ploug et al., 1998). Therefore, it has been proved that *GLUT4* translocation can fully explain the increased glucose transport in the membrane during exercise‐induced muscle contraction (Garippa et al., 1994). Taken together, these results suggest that exercise training subtly and effectively increases and coordinates the expression levels of fundamental genes involved in a wide range of processes related to muscle energy production and metabolism.

During physical exercise or activity, the increase in sympathetic outflow destroys lipid homeostasis, and then increases the demand of muscle for substrate by inducing lipolysis pathway and inhibiting fatty acid synthesis (Bosma, 2014; Koh et al., 2007). Indeed, our results are in line with the latter statement, with a corresponding decrease in intramuscular fat content and in the level of some unsaturated fatty acids with exercise training. Unsaturated fatty acid metabolic process (BP GO:0,033,559), arachidonic acid metabolic process (BP GO:0,019,369), and fatty acid derivative metabolic process (BP GO:1,901,568) GO ontologies (Figure 2) were also overrepresented among up‐regulated expression genes following exercise. However, our results also show that among the differentially expressed genes, there are some genes related to lipid metabolism, such as *DGAT2*, *ACSF3*, *OLR1*, *GPCPD1,* and *PLIN1* (Table S2), that are involved in the regulation of lipid storage or in fatty acid synthesis, which is contrary to the generally recognized exercise‐induced lipolysis pathway. The *DGAT* (diacylglycerol O‐acyltransferase) is a vital group of enzymes in catalyzing triacylglycerol biosynthesis and D. Li et al. (2020) found that *DGAT2*, which is related to energy metabolism, regulates IMF deposition in chicken. *ACSF3* (Acyl‐CoA synthetase family member 3, transcript variant X1) in mammals uses free malonic acid as substrate to synthesize malonyl‐CoA in mitochondria, which provides chain extender units for mitochondrial fatty acid synthesis (Witkowski et al., 2011). *OLR1* (oxidized low‐density lipoprotein receptor 1) can bind and degrade oxidized low‐density lipoproteins, and is expressed in a variety of cell types and closely linked to obesity (Nomata et al., 2009). Zambonelli et al. (2016) have demonstrated that *OLR1* is highly expressed in fat pigs compared with lean animals. Moreover, the SNPs in *OLR1* was also confirmed to be significantly associated with economic traits, such as marbling score in beef (Fonseca PD, 2015; Kaneda et al., 2011). *PLIN1* (perilipin 1) is localized to lipid droplets and is the most abundant protein associated with lipid droplets in adipocytes. It promotes triacylglycerol synthesis under basal and fed conditions and, in contrast, stimulates lipolysis during fasting or extended exercise (Brasaemle et al., 2009). Despite *PLIN1* being supposed to promote lipolysis and provide energy in the present trial, it became down‐regulated after 3 months of exercise training. In addition, C14:0, C14:1, and C18:0 content increased in the exercise group. A possible explanation of these results is that after the training, intramuscular fat and some fatty acids decreased significantly, which may induce a means of adaptive response to reduce lipolysis activation, to avoid long‐term movement and lack of nutrition supply Mongolian sheep excessive consumption of lipid and fatty acids decomposition. This is consistent with the study of de Souza Cordeiro et al. (2019), who observed significant weight loss in rats after training, but they also observed a decreased expression of *PPARα* and *CPT1*, which may induce an adaptive response to reduce the activation of oxidative pathways and thus avoid excessive fat weight loss in nonobese rats. Seideman et al. (1982) pointed out that the increased energy requirements of exercise must be met by increasing feed intake, not by reducing fat content. Coyle et al. (1997) found that the intake of carbohydrates before exercise can significantly reduce the oxidation of triglycerides and the mobilization of free fatty acids in the plasma, further reducing fat oxidation. This once again emphasizes that if you want to increase the amount of physical exercise to promote muscle metabolism to achieve the purpose of improving meat quality, you have to pay attention to nutritional supplements, especially carbohydrate intake before exercise.

Interestingly, there are two up‐regulations of KEGG pathways, the TNF signaling pathway and *IL‐17* signaling pathway (Figure 3). Previous studies have shown that lipolysis in adipose tissue is also regulated by proinflammatory cytokines and the TNF‐α and *IL‐6* in exercising muscle stimulate lipolysis through different pathways (Botion et al., 2001; M. Kelly et al., 2004; Langin & Arner, 2006; Ostrowski et al., 1998). Recent studies have emphasized that adipose tissue inflammation is reshaping the appropriate organization (including quantitative and qualitative modification in resident adipose tissue cells) necessary for proper control necessary for the expansion of adipose tissue, so these cytokines are associated with the biological control of intermediary metabolism (Asterholm et al., 2014; Choe et al., 2016; Menezes‐Garcia et al., 2014). Our results showed that, among the genes involved in the TNF and the IL‐17 signaling pathway, *SOCS3* (suppressor of cytokine signaling 3) is also involved in a number of GO terms related to muscle metabolism and development, such as lipid metabolism, developmental process, and regulation of phosphate metabolic process. In addition, the roles of *PTGS2* (prostaglandin‐endoperoxide synthase 2), *FOS* (Fos proto‐oncogene, AP‐1 transcription factor subunit), *TNFAIP3* (TNF alpha‐induced protein 3, transcript variant X1), and *MAP2K6* (mitogen‐activated protein kinase 6, transcript variant X1) in oxidative stress pathways, cellular development, and skeletal muscle growth have also been demonstrated (Almada et al., 2021; Li et al., 2018; Meng et al., 2017; Regassa & Kim, 2015). Fujishiro et al. (2001) indicated that the activation of *MAP2K6 and p38MAPK* signaling pathways could regulate the expression of glucose transporter and had an inhibitory effect on insulin signaling pathway. Therefore, we suggest that the increase in proinflammatory cytokines in muscle tissue induced by exercise training may be an effective stimulant of muscle metabolism remodeling. Nevertheless, additional research is required to better explain the effects of these cytokines on muscle metabolism and development in exercise‐induced skeletal muscle of Mongolian sheep, and these cytokines could be evaluated as candidate genes for improvement of meat quality.

## 5 CONCLUSION

Three months of exercise training effectively induced adaptive responses, and increased genes and pathways involved in muscle metabolism and development as well as oxidation‐reduction process were observed to satisfy increased metabolic demands during physical exertion. Physical exercise in sheep caused meat with higher shear force, and lower intramuscular fat, and color parameters, L*, a*, and b* than control group with moderate differences in the fatty acids profile and carcass characteristics. Therefore, the exercise regimen used in this study did not provide an advantage in terms of carcass yield and meat quality and induced another adaptive response in muscle tissue to lipid synthesis to counter excessive nonphysiological energy expenditure. Our results provide insights into the mechanisms by which exercise training affects quality traits and fatty acid composition in Mongolian sheep and will contribute to a better understanding of the biological and molecular processes underlying muscle physiology and meat quality.

## CONFLICT OF INTEREST

The authors state that there is no conflict of interest and financial, personal, or other relations with others or organizations.

## AUTHOR CONTRIBUTIONS

MZ, YYG, and RNS conceived and designed the experiments. JLL, HH, YZ, and DY performed the experiments. MZ analyzed the data. YYG, YJ, LS, and LHZ contributed reagents/materials/analysis tools. MC designed the study. MZ wrote the article.

## Supporting information

Table S1

Table S2

Table S3

Table S4

Table S5

Table S6

## Data Availability

All sequencing data are available through the NCBI Sequence Read Archive under the accession number PRJNA744852. The raw reads of our transcriptome data have been deposited into the NCBI Short Read Archive (SRA, http://www.ncbi.nlm.nih.gov/sra/) under accession number SRP327950.
