# Erratum for effects of physical exercise on muscle metabolism and meat quality characteristics of Mongolian sheep

**DOI:** 10.1002/fsn3.2996

**Published:** 2022-11-03

Zhang, M., Guo, Y., Su, R., Corazzin, M., Li, J., Huang, H., Zhang, Y., Yao, D., Su, L., Zhao, L., & Jin, Y. (2022). Effects of physical exercise on muscle metabolism and meat quality characteristics of Mongolian sheep. Food Science & Nutrition, 10, 1494–1509. https://doi.org/10.1002/fsn3.2768

In the article by Zhang et al., the funding information was incomplete and should have appeared as:

The Special Project of Scientific and Technological Achievements Transformation in Inner Mongolia Autonomous Region (2019CG066), Major Special Projects of Natural Science Foundation in Inner Mongolia Autonomous Region (2020ZD11), The National Nature Science Foundation of China (32160589), the First‐rate Discipline Construction Project of Food Science and Engineering in Inner Mongolia Autonomous Region (SPFC202004), Natural Science Foundation of Inner Mongolia (2018MS03054).

